# Therapeutic potential of stem cells expressing suicide genes that selectively target human breast cancer cells: Evidence that they exert tumoricidal effects via tumor tropism

**DOI:** 10.3892/ijo.2012.1523

**Published:** 2012-06-20

**Authors:** BO-RIM YI, KELVIN J. CHOI, SEUNG U. KIM, KYUNG-CHUL CHOI

**Affiliations:** 1 Laboratory of Veterinary Biochemistry and Immunology, College of Veterinary Medicine, Chungbuk National University, Cheongju, Chungbuk, Republic of Korea;; 2 Departments of Chemistry and; 3 Medicine, University of British Columbia, Vancouver, British Columbia, Canada;; 4 Medical Research Institute, Chung-Ang University College of Medicine, Seoul, Republic of Korea

**Keywords:** gene-direct enzyme/prodrug therapy, stem cell therapy, cytosine deaminase, carboxyl esterase, thymidine kinase, breast cancer

## Abstract

Breast cancer is the most prevalent cancer in women worldwide and is classified into ductal and lobular carcinoma. Breast cancer as well as lobular carcinoma is associated with various risk factors such as gender, age, female hormone exposure, ethnicity, family history and genetic risk factor-associated genes. Genes associated with a high risk of developing breast cancer include *BRCA1*, *BRCA2*, *p53*, *PTEN*, *CHEK2* and *ATM*. Surgery, chemotherapy, radiotherapy and hormone therapy are used to treat breast cancer but these therapies, except for surgery, have many side-effects such as alopecia, anesthesia, diarrhea and arthralgia. Gene-directed enzyme/prodrug therapy (GEPT) or suicide gene therapy, may improve the therapeutic efficacy of conventional cancer radiotherapy and chemotherapy without side-effects. GEPT most often involves the use of a viral vector to deliver a gene not found in mammalian cells and that produces enzymes which can convert a relatively non-toxic prodrug into a toxic agent. Examples of these systems include cytosine deaminase/5-fluorocytosine (CD/5-FC), carboxyl esterase/irinotecan (CE/CPT-11), and thymidine kinase/ganciclovir (TK/GCV). Recently, therapies based on genetically engineered stem cells (GESTECs) using a GEPT system have received a great deal of attention for their clinical and therapeutic potential to treat breast cancer. In this review, we discuss the potential of GESTECs via tumor tropism effects and therapeutic efficacy against several different types of cancer cells. GESTECs represent a useful tool for treating breast cancer without inducing injuries associated with conventional therapeutic modalities.

## Contents

1. Breast cancer
2. GEPT using various prodrug/enzymes
3. Genetically engineered stem cell (GESTEC)-based therapy for treating breast cancer
4. Conclusions

## 1. Breast cancer

### Overview

Breast cancer is the most frequently diagnosed cancer and the leading cause of cancer mortality in women, accounting for 23% of all cancer cases and 14% of all cases of cancer mortality (1). The breasts are composed of fat, glandular, and connective (fibrous) tissues, and contain several lobes which are divided into lobules that end in milk glands. Tiny ducts run from the glands, converge, and end in the nipple. Breast cancer changes the size or shape of the breast and can be separated into two histopathological categories: ductal and lobular carcinoma (2). Additionally, these carcinomas are further divided into *in situ* and invasive carcinomas according to whether the tumor is confined to the glandular component of the organ or whether it has invaded the stroma (3). Ductal carcinoma represents 80% of breast cancer cases and presumably originates from malignant epithelial cells within the ducts or tubes that carry milk to the nipple from the breast (4). Lobular carcinoma is a less common form of breast cancer that commences in the milk-producing lobules of the breast (5). This type of carcinoma is composed of acini filled with a small, round, polygonal or cuboidal cells (6).

Breast cancer progression includes five stages defined according to tumor size, spread to the lymph nodes, and metastasis (spread to a more distant part of the body) (7). Stage 0 is a pre-cancerous state in which the cancerous cells have not spread outside of the milk-producing lobules or ducts. Lesions in this stage are also referred to as ductal carcinoma *in situ* (DCIS) and lobular carcinoma *in situ* (LCIS) (8). DCIS is generally categorized into the five most common architectural subtypes, including papillary, micropapillary, cribriform solid, and comedo (9). Stages I to III are characterized by lesions within the breast or regional lymph nodes; these stages are based on the size of the tumor and the spread to the lymph nodes (10). Finally, stage IV is metastatic cancer that has spread to other organs of the body (i.e., lungs, bones, liver, or brain) (11). Although breast cancer is the most frequently diagnosed cancer and the leading cause of cancer mortality in women, if detected during the early stages it can be treated successfully by surgery or chemotherapy (12).

### Causes

Variable risk factors for breast cancer include gender, age, female hormone exposure, ethnicity, obesity, family history of breast cancer, genetic risk factors, and many abnormal conditions of the breast (13,14). Being female is the main risk factor for developing breast cancer since women have significantly more breast cells than men. Nevertheless, men can also develop breast cancer but they account for <1% of all breast cancer cases (15). Clinically, breast cancer in men is similar to that in women and is also affected by hormonal, genetic, and environmental factors (16).

In women, cells in the breast are exposed to growth-stimulating female hormones including estrogen (E2) and progesterone (P4) (17). E2 stimulates breast cell division which can increase the risk of permanent DNA damage (18). The growth factor transforming growth factor-α (TGF-α) can also affect cell division, and overexpression of this factor is associated with increased cell division in breast cancer (19).

The risk of developing breast cancer increases with age and doubles every 10 years until menopause (20). Age is the strongest risk factor for breast cancer after gender (21). There are also numerous genetic risk factors for breast cancer. Numerous cases of cancer begin when one or more genes in a cell mutate, thereby producing an abnormal protein or no protein at all (22). Production of an abnormal protein and lack of protein production may cause cells to divide uncontrollably and become cancerous (23). The normal function of genetic risk factor-associated genes is the suppression of tumorigenesis. Genes associated with a high risk of developing breast cancer include *BRCA1*, *BRCA2*, *p53*, *PTEN*, *STK11*, *CHEK2*, and *ATM*(24,25). Finally, various other factors such as medical history, life style, dense breast tissue, alcohol intake, and smoking can promote the development of breast cancer (26).

### BRCA1 and BRCA2

Mutation of breast cancer type 1 and 2 susceptibility proteins (BRCA1 and BRCA2) cause most hereditary breast or ovarian cancer syndromes. *BRCA* gene-associated mutations might also be caused by Li-Fraumeni-like syndrome (LFS) (27,28). Mutation of these genes confers a 43–84% risk of breast cancer by the age of 50–70 in women (29,30). It is now clear that the normal protein products of BRCA1 and BRCA2 are tumor suppressors (31). *BRCA1* is located on chromosome 17. The BRCA1 protein acts as a hub protein that promotes genomic stability and DNA repair by its involvement in homologous recombination and nucleotide excision repair, DNA damage response and cell cycle check point control, chromatin remodeling, transcriptional regulation, and protein ubiquitylation (32). *BRCA2* is located on chromosome 13. The BRCA2 protein plays an important role in maintaining genomic stability via homologous recombination, both during meiosis and repair of double-strand breaks (33). Both BRCA1 and BRCA2 mutations have been found more often in patients with high grade breast cancer compared to age-matched control patients (34). However, tumors with BRCA1 mutations have high mitotic counts and ones with BRCA2 mutations mostly contain less tubular structures. Furthermore, BRCA2 mutations are associated with a more extensive intraductal component than BRCA1 mutations, and increase the risks for certain childhood tumors (35).

### p53

*p53* is a known tumor suppressor gene encoding a sequence-specific transcriptional regulator which controls cell cycle progression, senescence, differentiation, DNA repair, and apoptosis (36). This gene has a major function in responding to cellular stress factors, such as DNA damage and hypoxia, resulting in a cascade of events that reduces the risk of cancer and prevents tumor development (37). Moreover, *p53* mutations have been observed in all the major histogenetic groups, including cancer of the colon, stomach, breast, ovary, and esophagus, and account for >50% of all cancer cases (38). A point mutation in the *p53* gene has been found between exon 4 and 10 that is located within the DNA binding domain of the p53 protein (39). A somatic mutation in the *p53* gene is the most common genetic change found in 20–35% of breast cancers and is associated with poor prognosis (40,41). A significant number of breast cancer cases is linked with *BRCA1* mutations that may affect p53 function, and activates a p53-dependent response (42). Furthermore, high expression of p53 is more frequently found in estrogen and progesterone receptor-negative breast cancers (43,44) and is also associated with a high proliferation rate, high histological grade, aneuploidy, and decreased survival rates (45).

### PTEN

Phosphatase and tensin homolog (PTEN) encoded by the *PTEN* gene in humans has been identified as a tumor suppressor in many types of cancer (46). This lipid phosphatase is involved in cell cycle regulation and prevents cells from growing and dividing too rapidly (47). The phosphatidylinositol (3,4,5)-trisphosphate (PIP3) kinase-protein kinase B (PI3K-AKT) pathway is activated in human cancers. Activated PI3K is phosphorylated phosphatidylinositol-4,5-bisphosphate (PIP2) to generate PIP3 (48,49). PIP3 activates Akt and is an important lipid second messenger that has a role in tumorigenesis (50). On the other hand, the PTEN protein, the key agonist of PI3K-AKT signaling, is inactivated in a broad spectrum of human cancers (51). However, somatic PTEN deletions and mutations have been observed in breast, brain, prostate, and kidney cancer cell lines as well as in several primary tumors such as endometrial carcinomas, malignant gliomas, melanomas, and thyroid tumors (52,53). In particular, Cowden syndrome patients have a germ line PTEN mutation, and an increasing amount of data have associated PTEN loss with breast cancer (5–21%) (54). It has also been reported that suppression of PTEN function increases breast cancer chemotherapeutic drug resistance (55,56). Some cell lines with mutated PTEN have an abnormal cell cycle and defective apoptotic responses (57).

### CHEK2 and ATM

The product of the cell cycle checkpoint kinase 2 (*CHEK2*) gene responds to DNA damage (double-strand breaks) in a dynamic, multistep process and protects genomic integrity (58). CHEK2 is a serine/threonine protein kinase found in humans and yeast. Activation of CHEK2 is regulated through phosphorylation by ataxia telangiectasia mutated (ATM) in both yeast and humans (59). ATM belongs to the PI3K-related protein kinase (PIKK) family and is responsible for the immediate and rapid response to double-strand breaks (60,61). However, mutation of this gene causes the development of ataxia-telangiectasia (AT), a neurodegenerative disease (62). The relationship between AT and breast cancer was first reported 20 years ago with the observation that relatives of AT patients have an increased risk of breast cancer (63).

Activated CHEK2 phosphorylates critical cell cycle proteins that results in the stabilization of p53 and the inhibition of Cdc25C phosphatase, leading to G1 cell-cycle arrest along with the prevention of entry into mitosis and the activation of DNA repair (64). This kinase also phosphorylates BRCA1, resulting in get back DNA damage (65). Mutations in the *CHEK2* gene, including truncation variant 1100delC, have been reported to increase breast cancer risk by up to two-fold and may vary according to the Li-Fraumeni syndrome as well as breast cancer (66). Susceptibility to cancer due to this gene variation was first described in 1999, and the products of the *CHEK2* and *ATM* genes are now known to be involved in p53 inactivation (67).

### Breast cancer treatments

Breast cancer is almost always treated with surgery, chemotherapy, radiotherapy, and hormone therapy. Surgical procedures, called mastectomy or lumpectomy, have a role in treating most patients with breast cancer (68). During these procedures, the cancerous lesions are removed from the breast along with some of the surrounding tissue. For this reason, the number of patients who receive breast implants after undergoing a mastectomy has increased (69). After performing surgery to treat breast cancer, radiation is used as an adjuvant treatment depending on the disease stage (70).

Hormonal therapy, including administration of tamoxifen, raloxifene (a selective estrogen receptor modulator, SERM), and aromatase inhibitors (AIs), increases the survival rate of hormone-sensitive breast cancer patients (71). Treatment of breast cancer patients with AIs is more effective than tamoxifen although patients receiving AIs have a higher prevalence of osteoporosis, bone fractures, and musculoskeletal symptoms, particularly joint pain and stiffness (72).

Chemotherapy is given to slow or stop the growth of cancer cells. For this, 5-fluorouracil (5-FU), cyclophosphamide, methotrexate, anthracyclines, trastuzumab, and taxanes are primarily used (73). If the breast cancer is positive for human epidermal growth factor receptor 2 (HER-2), it is treated with trastuzumab (herceptin) which targets the *HER-2* oncogene (74). 5-FU has also been the preferred chemotherapeutic agent for treating a majority of solid tumors, including gastric and colon cancers (75). However, serious side-effects such as alopecia, anesthesia, diarrhea, and arthralgia, as well as high dose requirements have limited the use of these chemotherapeutic agents (76).

## 2. GEPT using various prodrug/enzymes

Gene-direct enzyme/prodrug therapy (GEPT), or suicide gene therapy, aims to improve the therapeutic efficacy of conventional cancer radio- and chemotherapy without side-effects (77,78). This system is a novel approach with the potential to selectively eradicate tumor cells (79). For this, an exogenous suicide enzyme gene is delivered to tumor cells (80). GEPT systems most often involve the use of a viral vector (adeno-, lenti-, or retroviral vectors) to deliver a gene not normally found in mammalian cells that produces enzymes which, when expressed, can convert a relatively non-toxic prodrug into a toxic agent (81,82).

GEPT systems involve two separate events: direct cell death and cell death via the bystander effect (83). The viral vectors transfected into the target tumor cells induced cell death (84). Direct cell death is caused by expression of the viral DNA in the targeted tumor cells (85). Next, cell death via the bystander effect is induced by the gene transfer of a viral or bacterial enzyme into targeted tumor cells. The enzymes convert an inactive prodrug into a short-lived toxic metabolite, leading to the death of cells surrounding the targeted tumor cells (86). Prodrugs can be defined as pharmacologically inactive derivatives which require chemical transformation for the release or conversion into the active drug (87). A suicide enzyme converts the administered non-toxic prodrug into an active drug which subsequently kills tumor cells but not normal tissues (88). Several types of suicidal genes have been studied and used for therapeutic purposes (82).

An advantage of these GEPT systems derives from the local bystander effect through which more wide-spread cell death is achieved without the need to express the gene in all cells (89). This is due to the ability of the toxic metabolite to diffuse freely across cells membranes or via gap junctions (90). Currently, a large number of enzyme/prodrug systems have been developed for GEPT. These include the cytosine deaminase/5-fluorocytosine (CD/5-FC), carboxyl esterase/irinotecan (CE/CPT-11), and thymidine kinase/ganciclovir (TK/GCV) systems (91).

### Cytosine deaminase/5-fluorocytosine (CD/5-FC)

One of the most widely used suicidal genes is bacterial or yeast CD (from *Escherichia coli* or *Saccharomyces cerevisiae*) (92). Both bacterial and yeast CD have been shown to inhibit tumor growth (93). The enzyme encoded by the *CD* gene catalyzes the conversion of cytosine into uracil; it is an important member of the pyrimidine salvage pathway in prokaryotes and fungi, but is not present in multicellular eukaryotes (mammalian cells) (94,95). 5-FC is a hydrophilic antifungal drug with low toxicity in humans (96). CD catalyzes the conversion of the non-cytotoxic prodrug 5-fluorocytosine (5-FC) into the cytotoxic chemotherapeutic agent 5-FU, resulting in anti-tumor activity (97). CD is currently being explored for use in gene therapy applications against solid tumors due to this activity (98).

The CD/5-FC system is very effective for treating human cancers as non-toxic 5-FC systemically administered can be converted into the cytotoxic agent 5-FU by the *CD* gene product located in the vicinity of the cancer (99). Deamination of the 5-FC prodrug by CD results in the formation of two toxic metabolites: 5-fluorodeoxyuridine monophosphate (FdUMP) and 5-fluorouridine triphosphate (FURTP). FdUMP is a potent inhibitor of thymidylate synthetase (TS) which is an enzyme essential for DNA synthesis. This compound impairs DNA synthesis and promotes apoptosis in bacteria and tumor cells (100,101).

### Carboxyl esterase/irinotecan (CE/CPT-11)

CE enzyme is a serine esterase found in a variety of tissues from numerous mammalian species (102). This enzyme plays a critical role in increasing the solubility and bio-availability of therapeutic agents (103,104). It is cleaved into the bulky piperidino sidechain of 7-ethyl-10-[4-(1-piperidino)-1-piperidino] carbonyl-oxycamptothecin (irinotecan or CPT-11). The anti-cancer agent CPT-11 is a prodrug that is activated by CE to generate the active form 7-ethyl-10-hydroxycamptothecin (SN-38) (105,106). SN-38 is a strong mammalian topoisomerase I inhibitor that is 1,000-fold more potent than CPT-11. This agent induces the accumulation of double-strand DNA breaks in actively dividing cancer cells (107).

### Thymidine kinase/ganciclovir (TK/GCV)

The most common GEPT uses the herpes simplex type-1 thymidine kinase enzyme (HSV-TK) in conjunction with a variety of guanosine-based prodrugs, compounds originally developed as antiviral agents (108). The HSV-TK enzyme converts to the prodrug into its monophosphate form, GCV, which is then further converted into the toxic triphosphates form, an intermediary metabolite, by cellular enzymes (109,110). These actions cause cell death by inhibiting the incorporation of dGTP into DNA without preventing progression through the S-phase; chain elongation is also inhibited (111).

## 3. Genetically engineered stem cell (GESTEC)-based therapy for treating breast cancer

Toxicity of anticancer agents to normal cells is a major limitation of breast cancer therapy (112). Therefore, stem cells have recently received a great deal of attention for their clinical and therapeutic potential to treat breast cancer. Stem cells are capable of continuous self-renewal and differentiation (113,114). A variety of stem cells, such as neural stem cells (NSCs), neural progenitor cells, and mesenchymal stem cells (MSCs) from bone marrow or adipose tissue, have been found to exert tumor-tropism effects (115). This ability makes these cells attractive for use as targeted delivery vectors for antitumor therapies (87,88,99,116–117). The tumor-tropism effects of stem cells are mediated by multiple cell-surface and secreted proteins, and candidate cytokines/receptors including stromal cell-derived factor-1 (SDF-1)/CXCR4, stem cell factor (SCF)/c-Kit, hepatocyte growth factor (HGF)/Met, vascular endothelial growth factor (VEGF)/VEGF receptor (VEGFR), monocyte chemoattractant protein-1 (MCP-1)/CCP, and high-mobility group box1 (HMGB1)/RAGE (87,88,99,117,118). In addition, NSCs appear to migrate to cancer cells more efficiently compared to MSCs. Although both NSCs and MSCs have a tumor tropic effect, NSCs (50–100% of total cell number) were proven to display greater tropism towards tumor cells than MSCs (40–75% of total cell number) (119).

**Figure 1:**
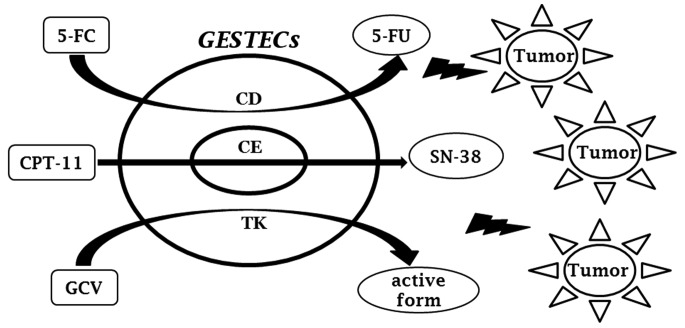
Schematic of genetically engineered stem cell (GESTEC)-based therapy. GESTECs are immortalized by retroviral vectors and contain suicide genes such as ones encoding cytosine deaminase (CD), carboxyl esterase (CE), and thymidine kinase (TK). CD converts the prodrug 5-fluorocytosine (5-FC) into a toxic agent, 5-fluorouracil (5-FU), which inhibits RNA and DNA synthesis. CPT-11 is a prodrug converted by CE into its active form, SN-38, which is a potent mammalian topoisomerase I inhibitor. Finally, the TK gene is found in herpes simplex virus (HSV). The product of this gene converts non-toxic ganciclovir into its toxic active form. These GESTECs exert tumor-tropism effects and may be used to treat several types of tumors.

The field of NSC research in recent years has seen major advances and efforts have been made to develop their use in potential stem cell-based transplantation therapies (120). NSCs can be used to generate all major mature neural cell types such as neurons, oligodendrocytes, glial cells and cells of neuronal lineages (121). The fetal brain, characterized by active neurogenesis, is thought to be a promising source of therapeutic NSCs (122). Previous studies have shown that NSCs derived from human fetal telencephalon can be used for GESTEC-based therapy for treating several different cancers as well as brain diseases (87,88,99). As this is based on a GEPT system, it involves the expression of several suicide enzymes (Fig. 1). In previous studies, GESTECs were immortalized by using retrovirus v-myc and suicide genes such as *CD*, *CE*, and *TK*. Therapeutic efficacy has been assessed by monitoring tumor-tropism in a brain cancer animal model (123).

In other studies, NSCs expressing *CD* or *CE* genes in an animal model of breast cancer brain metastasis were found to significantly reduce breast tumor mass in the brain (124). This demonstrated the therapeutic efficacy of GESTECs in the presence of a prodrug (114). Brain metastases originate from cells that do not reside in the brain. This suggests that breast tumor metastases in the brain attract GESTECs as well as the original brain tumor. The therapeutic efficacy of GESTECs for treating several other types of cancer cells (i.e., ovarian, endometrial, and lung cancer cells) as well as brain tumors, including medulloblastomas and gliomas has also been demonstrated *in vitro*. Furthermore, therapies using GESTECs may use as breast cancer treatment *in vitro* and *in vivo*.

## 4. Conclusions

Breast cancer is the leading cause of cancer related mortality among women worldwide. Several gene mutations lead to the development of breast cancer including ductal and lobular breast carcinoma. Chemo-, hormone-, and radiotherapies are used to treat breast cancer but these therapies are associated with many side-effects. For this reason, GEPT systems have been examined as a novel anticancer therapeutic approach with the potential to selectively eradicate tumor cells. Prodrugs used for GEPT are primarily antimetabolites that require cell cycling (S phase) to induce cytotoxicity and are not active against normal cells. These systems may involve the use of NSCs which express suicide genes and have the ability to selectively migrate to tumors. In summary, GESTECs using GEPT systems may be an effective new modality for treating breast cancer as well as brain tumors without inducing injurious effects commonly associated with more conventional anticancer therapies.
